# Nursing Salaries at Royal Infirmary, Wigan

**Published:** 1920-03-06

**Authors:** Hedley Lucas

**Affiliations:** General Superintendent and Secretary.


*To the Editor of* The Hospital.

### Nursing Salaries at Royal Infirmary, Wigan.

*To the Editor of* The Hospital.

Sir,—The announcement in this week's Hospital *re* nursing-staff salaries here conveys an erroneous impression. It is not correct that those who receive £20 to £30 and £50 to £70 are to have a further £5 and £10 per annum respectively, but that probationers are now to receive a scale of £20 to £30 per annum, the yearly increments being £5; sisters are now to have £50 to £70 per annum, the yearly increments being £10. In future the two nurses who receive the highest percentage of marks at the examination at the end of the three years' training will be given the opportunity to take the C.M.B. certificate, or its equivalent in massage training, at the Infirmary's expense.—Yours faithfully,

Hedley Lucas,
General Superintendent and Secretary.

